# Agreement of Corneal Measurements With Swept-Source Optical Coherence Tomography (Anterion) and Scheimpflug (Sirius) Device in Eyes With Dermatochalasis

**DOI:** 10.7759/cureus.89802

**Published:** 2025-08-11

**Authors:** Hatice Kubra Sonmez, Metin Unlu, Cem Evereklioglu, Fatih Horozoglu, Osman Ahmet Polat, Hatice Arda

**Affiliations:** 1 Ophthalmology, Erciyes University Faculty of Medicine, Kayseri, TUR; 2 Ophthalmology, Maya Eye Hospital, Kayseri, TUR

**Keywords:** anterion, cornea, dermatochalasis, keratometry, scheimpflug-placido topography, sirius, swept-source optical coherence tomography

## Abstract

Introduction

This study aimed to evaluate the agreement between two advanced anterior segment imaging devices, Anterion (Heidelberg Engineering GmbH, Heidelberg, Germany) and Sirius (CSO, Florence, Italy), in measuring keratometric and pachymetric parameters in patients with moderate to severe dermatochalasis.

Methods

Keratometric and pachymetric measurements were obtained using Anterion and Sirius devices in patients with moderate to severe dermatochalasis. The agreement between the two devices was assessed by comparing anterior and posterior corneal keratometry and corneal thickness. Statistical analyses were performed to determine the significance of differences and correlations between the measurements.

Results

Statistically significant differences were observed between the two devices in anterior and posterior corneal keratometry, except for posterior astigmatism and corneal thickness. However, these differences were clinically insignificant(low refractive differences). The study found excellent agreement between Anterion and Sirius for most parameters, with strong correlations and minimal disparities in measurements. Posterior astigmatism showed moderate agreement but still exhibited a strong correlation between the devices.

Conclusion

Both Anterion and Sirius provide reliable keratometric and pachymetric measurements in patients with dermatochalasis, allowing for interchangeable use. Accurate preoperative evaluations are essential for cataract or refractive surgery, as dermatochalasis may affect corneal measurements.

## Introduction

Dermatochalasis is the drooping of the skin of the upper eyelid due to a loss of skin tone, often sagging toward the lid margin. While this condition primarily raises aesthetic concerns, in severe cases, it can lead to visual changes, particularly with bitemporal visual field defects. There are studies in the literature indicating that long-term changes in corneal thickness, higher-order aberrations, contrast sensitivity, and corneal topography may occur following upper eyelid blepharoplasty surgery [1-3]. Dermatochalasis may introduce inaccuracies in keratometric and biometric measurements due to the upper eyelid sagging, interfering with the path of imaging light. This interference can result in lid-induced artifacts, partial obstruction of the visual axis, or shadowing effects, potentially distorting the accurate representation of both anterior and posterior corneal surfaces. These mechanisms may differentially impact device performance depending on the underlying imaging technology (e.g., Scheimpflug-based systems versus swept-source optical coherence tomography (SS-OCT)). Moreover, centralization of corneal measurements plays a critical role in keratometry calculations, particularly since keratometric values used in biometric formulas are typically derived from the central 2.5-3 mm zone of the cornea. Dermatochalasis may alter this centralization in some devices due to redundant upper eyelid tissue crowding the visual axis. Therefore, assessing the agreement of corneal parameters between these two devices in patients with dermatochalasis is important for identifying potential measurement deviations attributable to lid anatomy and understanding their implications for surgical planning. The patient groups recommended for surgery due to dermatochalasis generally fall into the middle and older age categories. It is plausible to consider that the keratometric changes caused by dermatochalasis could also affect the measurements for cataract and refractive surgeries, which are commonly performed in this age group. The precision of keratometric and biometric measurements in these patients is crucial for the success of the surgery.

This study aims to evaluate the consistency of different anterior segment devices in corneal measurements in dermatochalasis and to shed light on which device has high reliability to use in refractive surgeries in this patient group.

Several devices capable of taking keratometric and biometric measurements are available. Scheimpflug-Placido topography systems are among the most frequently used methods in anterior segment measurements [4]. Sirius (CSO, Florence, Italy) is equipped with a Placido disc system integrated with a Scheimpflug camera, which allows for more accurate imaging of the corneal curvature [5]. Sirius is a measurement method that provides highly efficient results in the mapping of the anterior segment and the detection of some corneal pathologies (corneal refraction, astigmatism, anterior and posterior corneal surface refractions, anterior chamber depth, iridocorneal angle, aberrations, etc.). Sirius provides data such as anterior and posterior corneal keratometry, total corneal power, anterior chamber depth, pupil diameter, corneal thickness, and ectasia indices. In recent years, SS-OCT-based analysis systems have also become widespread. Anterion (Heidelberg Engineering GmbH, Heidelberg, Germany), a high-resolution, multimodal OCT system, has been shown in some studies to provide non-invasive, highly accurate evaluations of the anterior and posterior corneal surfaces [6]. Although anterior segment imaging methods introduced in recent years offer higher resolution and more accurate measurements, comprehensive studies demonstrating the superiority of newer-generation systems and methods using other technologies are still lacking. Differences between patient groups and their superiority in different pathologies remain a matter of ongoing investigation. High reliability is particularly important in practice for refractive interventions.

The aim of this study is to assess anterior and posterior corneal keratometry, astigmatism, and corneal thickness measurements in patients with dermatochalasis using two different anterior segment imaging methods (Sirius and Anterion).

## Materials and methods

This retrospective study was approved by the Clinical Research Ethics Committee of Erciyes University Faculty of Medicine, adhering to the principles of the Declaration of Helsinki (approval no: 2024/104). The study included 43 eyes of 22 patients presenting with moderate to severe dermatochalasis (preoperative). Exclusion criteria included eyelid abnormalities, a history of intraocular and refractive surgery, recent contact lens use, any corneal pathology, severe dry eye, and ectatic disorders.

All patients underwent a complete ophthalmologic examination. Best corrected visual acuity (BCVA) (Snellen decimal scale), intraocular pressure (mmHg), and levator function (mm) were also recorded. While patients with low refractive errors were included in the study, the clinical relevance of keratometric deviations obtained from the devices was evaluated based on the approximation that a 1 diopter (D) change in total keratometry corresponds to approximately a 1 D deviation in intraocular lens (IOL) power [7].

Measurements were performed without pupil dilation under the same lighting conditions and by the same technician. The imaging was completed within approximately the same timeframe, with about a five-minute interval between the two devices. Scan quality rating was assessed by the ophthalmologist based on corneal acquisition area, missing data, fixation quality, and lid artifacts. Each measurement was taken at least three times, and only the highest quality, acceptable images were included in the statistical analysis. The order of imaging for the patients included in the study was determined randomly. Data for each patient were evaluated by the same ophthalmologists (HKS, MU).

Sirius corneal topography consists of a small-angle Placido disc-based corneal topography device with a Scheimpflug camera capable of rotating 360°, capturing 22 rings and 25 radial sections of the cornea [5]. The rings provide data on elevation, slope, and curvature. Data for the anterior cornea are derived from both the Placido disc and Scheimpflug images, while information about the posterior cornea and iris is obtained from Scheimpflug images. The pachymetry map is created using data from both the anterior and posterior corneal surfaces, with an acquisition time of five to six seconds [5].

**Figure 1:**
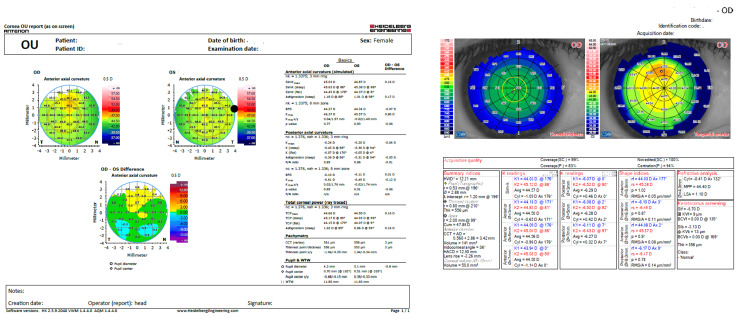
Sample displays Anterion (swept-source optical coherence tomography (OCT)) (left side) and Sirius (Scheimpflug-Placido) (right side) anterior and posterior K values, astigmatism magnitude, and corneal pachymetry metrics. Patient identifiers have been anonymized.

The Anterion device is an SS-OCT-based anterior segment imaging system. It provides topography of both the anterior and posterior corneal surfaces individually, documenting all refractive data for both surfaces, along with axial length [6]. Additionally, it performs biometric calculations using the refractive values of the anterior and posterior corneal surfaces. Measurements are made with reference to the corneal apex using the eye-tracking system. Representative measurements and outputs from both devices are illustrated in Figure 1. 

The common data from both devices were compared, including: mean anterior simulated axial curvature (simulated K (Sim K)), Sim K steep (K2), Sim K flat (K1), anterior and posterior astigmatism at 3-mm, mean posterior K, posterior K steep (postK2), posterior K flat (postK1), central corneal thickness (CCT), and thinnest point thickness of the cornea.

Statistical analysis

The data were analyzed using the Statistical Package for Social Sciences (SPSS) for Windows (Version 22.0; IBM Corp., Armonk, NY). Descriptive statistics exhibiting a normal distribution were expressed as mean ± standard deviation, while those not exhibiting a normal distribution were expressed as median, maximum, and minimum values. The Shapiro-Wilks test was used to assess normality, with a p-value of more than 0.05 indicating normal distribution. Differences in measurements between the two devices were evaluated using a one-sample t-test. The intraclass correlation coefficient (ICC) was calculated to assess the degree of agreement between the two devices. The ICC evaluation was made as follows: less than 0.50 is poor, between 0.50 and 0.75 is moderate, between 0.75 and 0.90 is good, and 0.90 and above is excellent. Pearson correlation was used to calculate the correlation coefficient for normally distributed data, while Spearman correlation was used for data that did not exhibit normal distribution. The agreement between values measured by the two devices was analyzed using a Bland-Altman plot and is expressed as 95% limits of agreement (LoA). p-values less than 0.05 were considered statistically significant. We performed a power analysis. Alpha (type I error) and beta (type II error) levels were set at 0.05 and 0.2, respectively. Effect size was set at 0.5. The number of eyes was estimated as 34.

## Results

Demographic and clinic status

The study included 43 eyes (22 right, 21 left) of 22 patients (five males, 17 females) with surgical indications for dermatochalasis. Both eyes of 21 patients were included, while one patient’s left eye was excluded due to previous intraocular surgery. The mean age was 52.37 ± 9.71 years. Three patients had a diagnosis of hypertension, while no additional comorbidities were present in the others. The mean BCVA was 0.97 ± 0.05, intraocular pressure was 15.46 ± 2.89 mmHg, and levator function was 15.39 ± 1.44 mm. Mild dry eye symptoms were present in 19% of the patients.

Comparison of keratometric and pachymetry parameters of two devices

The corneal anterior surface keratometric parameters were as follows: mean anterior simulated K (Sim K) (44.02 ± 1.37/43.80 ± 1.4), steep K (44.45 ± 1.44/44.15 ± 1.48), and flat K (43.61 ± 1.4/43.42 ± 1.41) (Anterion/Sirius), with statistically significant differences between the two devices (p < 0.001 for all). The largest difference was observed in steep K (mean difference ± SD 0.29 ± 0.36, 95% LoA -0.42 to 1.0). For the corneal posterior surface keratometric parameters, the mean posterior K was -6.15 ± 0.2/-6.23 ± 0.2, steep K was -6.30 ± 0.22/-6.41 ± 0.21, and flat K was -6.01 ± 0.19/-6.07 ± 0.21 (Anterion/Sirius), with statistically significant differences (p < 0.001 for all). Again, the largest difference was in steep K (mean difference ± SD 0.10 ± 0.08, 95% LoA -0.06 to 0.26).

There was a significant difference in anterior astigmatism between the two devices (mean difference ± SD 0.12 ± 0.3, p = 0.012), while no significant difference was found for posterior astigmatism (mean difference ± SD 0.03 ± 0.14, p = 0.17). Differences in CCT and thinnest CCT parameters were also not statistically significant (p = 0.69, p = 0.58, respectively) (Table 1).

**Table 1:** Comparison of mean keratometric measurements of the anterior and posterior corneal surface between the two devices *One-sample t-test. CCT: central corneal thickness; D: diopter; LoA: limits of agreement; SD: standard deviation; μm: micrometer

Parameter	Anterion (mean ± SD)	Sirius (mean ± SD)	Difference (mean ± SD)	95% LoA	p-value*
Mean anterior Sim K (D)	44.02 ± 1.37	43.80 ± 1.4	0.22 ± 0.27	-0.32-0.76	<0.001
Steep K (K2) (D)	44.45 ± 1.44	44.15 ± 1.48	0.29 ± 0.36	-0.42-1.0	<0.001
Flat K (K1) (D)	43.61 ± 1.4	43.42 ± 1.41	0.18 ± 0.3	-0.40-0.76	<0.001
Anterior astigmatism (D)	0.85 ± 0.61	0.73 ± 0.5	0.12 ± 0.3	-0.48-0.72	0.012
Mean posterior K (D)	-6.15 ± 0.2	-6.23 ± 0.2	0.08 ± 0.06	-0.04-0.2	<0.001
Steep K post (K2) (D)	-6.30 ± 0.22	-6.41 ± 0.21	0.10 ± 0.08	-0.06-0.26	<0.001
Flat K post (K1) (D)	-6.01 ± 0.19	-6.07 ± 0.21	0.06 ± 0.08	-0.10-0.22	<0.001
Posterior astigmatism (D)	-0.3 ± 0.12	-0.33 ± 0.17	0.03 ± 0.14	-0.25-0.31	0.17
CCT (μm)	537.95 ± 32.87	537.14 ± 33.55	0.81 ± 13.52	-25.6-27.3	0.69
Thinnest CCT (μm)	534.02 ± 32.74	532.95 ± 34.54	1.07 ± 12.62	-23.6-25.8	0.58

Compatibility between devices

Correlation analysis between the two devices showed a strong correlation for all parameters except for posterior astigmatism (Table 2). When assessing the consistency of the measurements using the ICC, excellent agreement was found for all parameters except posterior astigmatism, which demonstrated only moderate agreement (ICC = 0.68, r = 0.568), while excellent agreement was obtained for all other parameters (ICC > 0.9) (Table 2). The moderate ICC for posterior astigmatism may be partly due to the inherent challenges in imaging the posterior corneal surface. Factors such as lower reflectivity, increased measurement noise, and the sensitivity of the measurement algorithms, especially when using different imaging modalities (SS-OCT vs. Scheimpflug-Placido topography), might contribute to this observed variability. Despite these differences, the overall mean difference remained minimal and statistically non-significant (p = 0.17), indicating that the measurements are still clinically acceptable.

**Table 2:** Correlation comparison of measurements between two devices *Spearman’s correlation; ^§^Pearson correlation. CCT: central corneal thickness; D: diopter; ICC: Intraclass correlation coefficient; μm: micrometer

Parameter	ICC	95% CI		r	p-value^§,*^
		Lower	Upper		
Mean anterior Sim K (D)	0.984	0.14	0.31	0.963*	<0.001
Steep K (K2) (D)	0.974	0.18	0.41	0.958*	<0.001
Flat K (K1) (D)	0.985	0.09	0.27	0.954*	<0.001
Anterior astigmatism (D)	0.908	0.03	0.22	0.854*	<0.001
Mean posterior K (D)	0.937	0.06	0.1	0.860*	<0.001
Steep K post (K2) (D)	0.909	0.08	0.13	0.858*	<0.001
Flat K post (K1) (D)	0.935	0.04	0.09	0.790*	<0.001
Posterior astigmatism (D)	0.686	-0.01	0.07	0.568^§^	<0.001
CCT (μm)	0.958	-3.35	4.97	0.917^§^	<0.001
Thinnest CCT (μm)	0.964	-2.81	4.95	0.931^§^	<0.001

**Figure 2:**
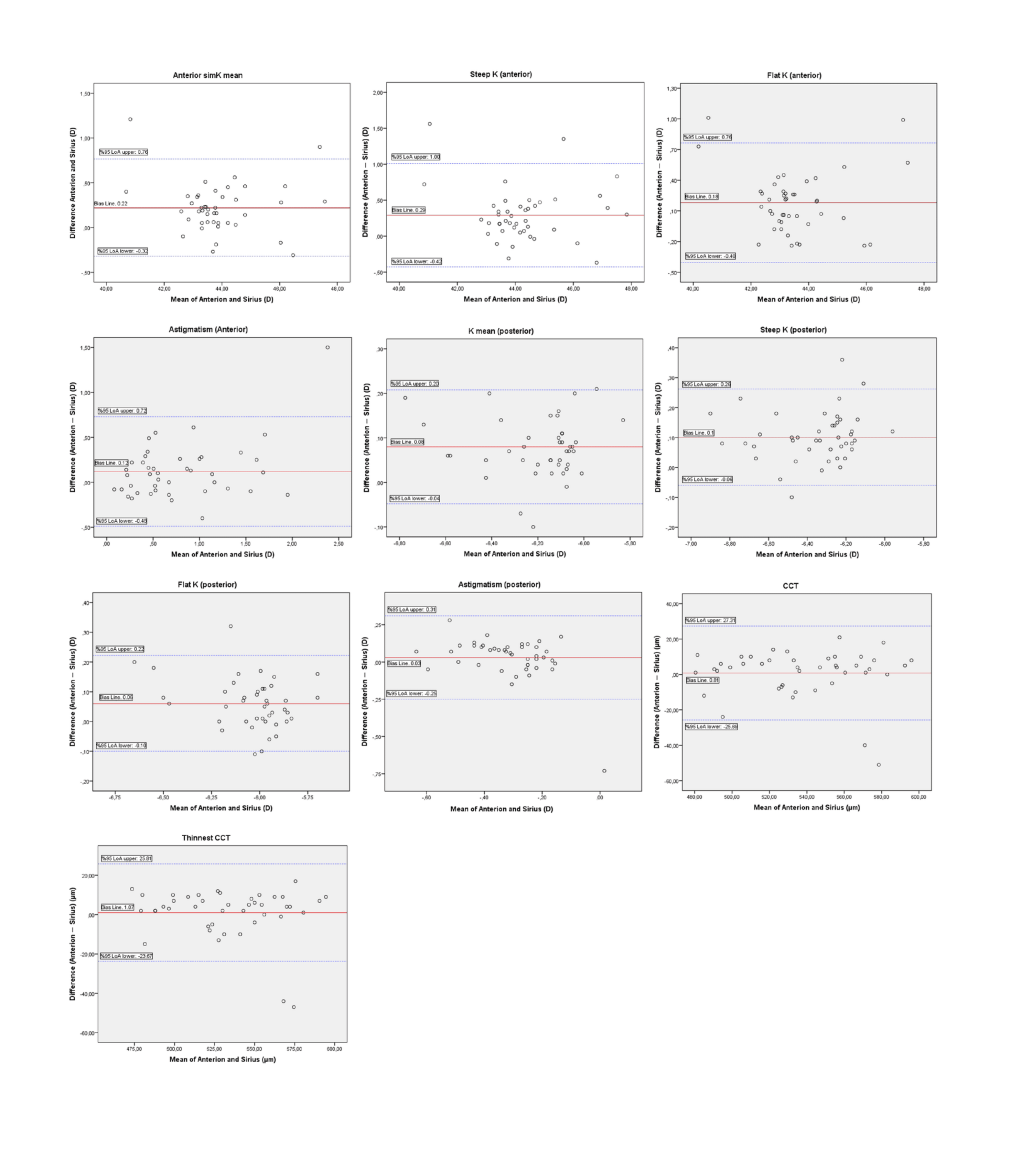
Bland-Altman plots showing agreement between keratometric and pachymetric measurements obtained from Sirius and Anterion devices. The x-axis shows the mean value between devices, and the y-axis shows the difference between devices (Anterion-Sirius). The mean bias (red continuous line) and the 95% width limits of agreement (blue dashed lines) are shown. For symmetry assessment, the distribution was interpreted as being evenly distributed around the bias; the widths (LoA) remained within clinically acceptable ranges. D: diopter; LoA: limits of agreement; SD: standard deviation; μm: micrometer

The Bland-Altman plots demonstrated suitable distribution for both keratometric and pachymetric measurements. The observed bias in Bland-Altman analyses is small, and the symmetric distribution of data on both sides suggests random variability rather than systematic bias in the measurements. The 95% LoA ranges are narrow, indicating consistent agreement between devices. Thus, excellent agreement was observed across all parameters (Figure 2).

## Discussion

In this study, we were the first in the literature to evaluate the agreement between two contemporary devices using different measurement techniques in patients with dermatochalasis. Although the patients included in the study had relatively high mean ages and good visual acuity, each was a candidate for cataract surgery in the near future. Therefore, the differences in measurements between devices could be important, especially in cases where blepharoplasty surgery is not desired and refractive or cataract surgery will be performed. Our results showed that, in this patient group, there were statistically significant differences in anterior and posterior keratometric measurements between the two devices, except for pachymetry and posterior astigmatism. However, these differences were small in magnitude and not clinically significant.

Altin Ekin et al. conducted a prospective study suggesting that dermatochalasis affects contrast sensitivity, refractive astigmatism, and higher-order aberrations [3]. In their study, patients who underwent upper eyelid blepharoplasty showed postoperative improvements in contrast sensitivity, reductions in astigmatism, and decreases in higher-order aberrations, with all changes being statistically and clinically significant. Similarly, other studies in the literature have demonstrated postoperative changes in visual fields, increases in contrast sensitivity, and alterations in astigmatism in this patient group [8-10]. Moreover, Zinkernagel et al. showed that the severity of blepharochalasis correlates with corneal topographic changes [11]. This underscores the importance of using the most sensitive method for preoperative evaluation in dermatochalasis patients who may undergo refractive surgery.

In a study comparing Scheimpflug imaging (Pentacam, OCULUS Inc., Arlington, WA) with Anterion for corneal aberrations, both devices were found to yield similar results in healthy eyes [12]. Other studies have shown that while posterior keratometric values differ between these two devices in healthy eyes, similar results are obtained for posterior astigmatism and pachymetric measurements [13,14]. Langenbucher et al. compared two SS-OCT-based devices, one of which was Anterion, for keratometric and biometric parameters, and found that Anterion obtained lower values for the posterior corneal surface [15]. They attributed this to the compensation of posterior surface image distortion caused by the reverse ray tracing used in OCT imaging. This difference may lead to lower total corneal power, which could result in variations in IOL calculations. Consistent with the literature, this study also found significantly lower posterior keratometric values with Anterion compared to Sirius. However, since the difference between the two devices was minimal, it was not considered clinically significant. This variation in measurements may be related to the technical differences in corneal surface measurements with the Anterion device. Pachymetric measurements were found to be similar and highly consistent between both devices.

In all keratometric and pachymetric measurements, except for posterior astigmatism, there was excellent agreement (ICC > 0.9 for all) and strong correlation (p < 0.001, r = 0.79 for posterior flat K; p < 0.001, r = 0.85 for other parameters) between the two devices. Although posterior astigmatism showed moderate agreement between the devices (ICC = 0.68), a strong correlation was observed (p < 0.001, r = 0.56). The comparatively lower ICC for posterior astigmatism, in contrast to the other parameters, may be attributed to several factors. The posterior corneal surface typically presents lower reflectivity and increased scattering, making it more challenging to image accurately [13]. Differences in the intrinsic imaging capabilities of SS-OCT (Anterion) and Scheimpflug imaging (Sirius) could lead to greater variability in this parameter. The processing algorithms used to derive posterior astigmatism may be more sensitive to small differences in corneal curvature. For instance, compensation techniques like reverse ray tracing in SS-OCT systems may introduce subtle discrepancies when compared to the methods employed by Scheimpflug devices [15].

Despite these challenges, the strong correlation (r = 0.56) and the minimal mean difference in posterior astigmatism (0.03 ± 0.14) suggest that while the absolute values may differ slightly between devices, the relative consistency is sufficient for clinical decision-making. In other words, even though the devices show moderate agreement in terms of ICC, they still reliably rank or compare posterior astigmatism values, allowing for their interchangeable use in preoperative evaluations. The Bland-Altman plots demonstrated excellent agreement across all parameters, with no significant discrepancies or wide LoA values. Therefore, in patients with moderate to advanced dermatochalasis, both methods can be used interchangeably for refractive measurements. Our findings suggest that temporal hooding, which is more prevalent in these patients, did not cause measurement differences between the devices, and similar results were obtained in corneal apex and 3-mm curvature measurements.

Patients with dermatochalasis frequently undergo blepharoplasty, and previous studies have demonstrated that upper eyelid surgery can lead to significant changes in corneal topography, astigmatism, and visual function. Therefore, it should be emphasized that preoperative measurements obtained in this study may change following blepharoplasty, potentially necessitating reassessment during surgical planning. Existing literature reports postoperative improvements in astigmatism and higher-order aberrations, suggesting that long-term follow-up, and if needed, inter-device comparisons can be beneficial in both refractive and IOL power planning [3,11]. Although both devices appear reliable for preoperative assessment, continued use of the same device postoperatively is advisable to maintain consistency in longitudinal monitoring.

This study had some limitations. One limitation was that all patients included had low refractive values. High spherical or cylindrical refractive values were excluded because they could have affected the assessment, but including such cases would have been useful to determine whether the devices maintain accuracy in these patients as well. Another limitation of the study is the exclusion of axis-specific and vectorial analysis of astigmatism, which particularly limits the interpretation of posterior astigmatism and its implications for toric IOL planning. Evaluating only the magnitude of astigmatism may overlook axis misalignments, which could result in unexpected discrepancies in total astigmatism correction between devices. Future studies incorporating vectorial analysis of both anterior and posterior astigmatism axes may help capture subtler differences and refine astigmatism correction strategies. However, since biometric comparisons were not performed, pupil diameters were not included in the analysis. Small sample size and short follow-up are other limitations. Our study will serve as a precursor to future studies that will evaluate changes over a longer period with a larger patient population.

## Conclusions

In this study, keratometric and pachymetric measurements obtained using the Sirius and Anterion devices in patients with dermatochalasis were compared, revealing statistically significant but clinically acceptable differences. Excellent agreement and strong correlation were observed for all parameters except posterior astigmatism. These findings directly support surgical decision-making in patients with dermatochalasis, particularly those undergoing refractive or cataract surgery. Although minor differences between devices were observed, they are unlikely to cause significant deviations in target refractive outcomes. However, awareness of these discrepancies is recommended when planning toric IOL implantation or precise astigmatic correction. Furthermore, considering that upper eyelid surgery (blepharoplasty) may alter corneal measurements, postoperative re-evaluation using the same device is advisable for consistent follow-up. The study contributes uniquely to the literature by being the first to compare these devices specifically in a dermatochalasis population. Future research with larger cohorts, including patients with higher refractive errors, is warranted to further validate and expand upon these findings.
